# Not all ‘natural’ is safe: A naturally occurring glucagon-like peptide-1 mimetic associated with drug-induced hypersensitivity syndrome

**DOI:** 10.1016/j.jdcr.2026.02.034

**Published:** 2026-02-23

**Authors:** Angelina Xu, Ryan Chen, Shauna Rice, Colleen Gabel, Nicole Lewandrowski, Nutan Gowda

**Affiliations:** a UMass Chan Medical School, Worcester, Massachusetts; b Department of Dermatology, UMass Chan Medical School, Worcester, Massachusetts; c Department of Pathology, UMass Chan Medical School, Worcester, Massachusetts

**Keywords:** DIHS, GLP-1 mimetic supplement toxicity, *Gymnema sylvestre*, herbal supplement, severe cutaneous adverse reaction

## Introduction

Therapeutic potential of glucagon-like peptide-1 (GLP-1) receptor agonists, initially developed for glycemic control in type 2 diabetes, has expanded beyond metabolic disease, with a recent boom in prescription volume worldwide.^1^ In February 2024, obesity management drug prescriptions reached 1.5 million, accounting for 0.41% of all prescriptions that month.^2^

Weight loss supplements now account for over 40% of the FDA dietary supplement database, often without regulatory oversight or robust safety data.^1^ Many “natural” supplements claiming to work through the GLP-1 pathway contain botanical ingredients such as ginseng extracts, berberine, psyllium, and, in our patient's case, *Gymnema sylvestre* extract.^1^, ^2^, ^3^

We report a case of drug-induced hypersensitivity syndrome (DIHS) secondary to a *Gymnema*-derived GLP-1 mimetic, highlighting dietary supplements as potential triggers of severe cutaneous adverse reactions amid the growing use of unregulated weight loss products.

## Case presentation

A 59-year-old woman with a history of childhood eczema presented to the emergency department with a 2-day history of a pruritic eruption that began on the left shoulder and progressively spread to the trunk, face, and extremities. The eruption was accompanied by facial swelling, headaches, fatigue, intermittent chills, and fever (Tmax 100.8 F). She denied the use of new medications, changes in soaps or detergents, or recent infections. The day prior to presentation, her primary care provider had prescribed oral prednisone for presumed allergic dermatitis, with no improvement.

She was on oral duloxetine at a stable dose for 3 years. Upon further questioning, she reported that she had begun using a weight loss supplement about 2 weeks prior to rash onset. This was advertised as “GLP-1 agonist-like”, containing *G sylvestre* extract, green tea, and caffeine. This supplement was discontinued 2 days prior to presentation. She denied the use of other over the counter or herbal preparations.

**Fig 1:**
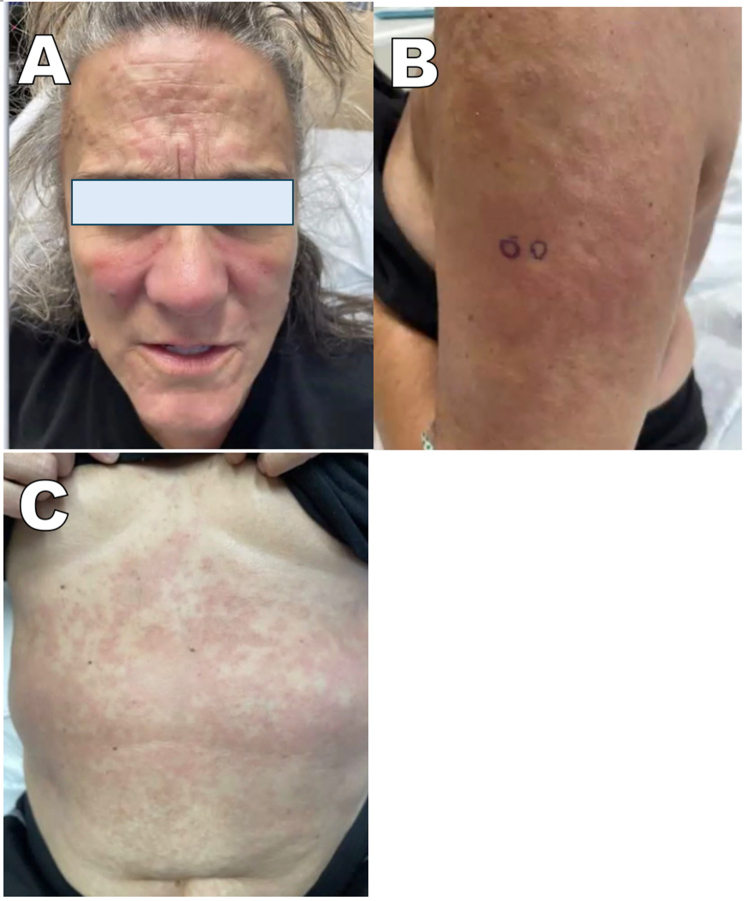
**A,** Indurated and edematous *pink papules* and plaques across cheeks and nose with notable facial edema. **B** and **C,** Confluent edematous papules and plaques on abdomen and lateral left arm, with locations marked for punch biopsy.

On examination, she had a diffuse morbilliform eruption involving the face, arms, and trunk (Fig 1), with a malar distribution across cheeks and nose. Approximately 55% of the total body surface area was involved. Marked cervical lymphadenopathy was present. There was no mucosal involvement.

**Fig 2:**
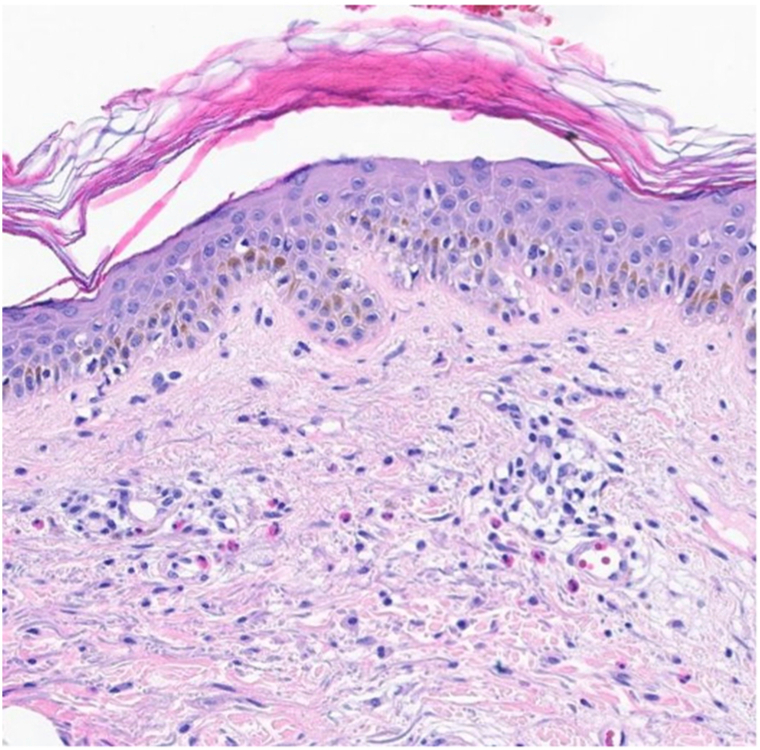
Punch biopsy demonstrated epidermal spongiosis, rare Langerhans cell microabscesses and underlying superficial perivascular and perifollicular lymphocytic infiltrate with numerous eosinophils. Direct immunofluorescence was negative.

Punch biopsy from the left arm revealed focal spongiosis of the epidermis with rare Langerhans cell microabscesses (Fig 2). In the superficial dermis, there was a perivascular and perifollicular lymphocytic infiltrate with numerous eosinophils. Direct immunofluorescence was negative for IgA, IgG, IgM, C3, and fibrinogen, arguing against an immunobullous disorder.

Initial labs revealed mildly elevated ALT (44 U/L, reference range (r/r) 10-40 U/L) and CRP (10.3 mg/L, r/r ≤ 9 mg/L), and normal renal function and urinalysis. Absolute eosinophils were normal on admission but rose to 21.1% (1.60 10∗3/uL, r/r 0.02-0.50 10∗3/uL) on hospital day 3. Tick panel, ANA, ANCA, and dsDNA was negative. Blood culture showed no growth. A high RegiSCAR score of 5 indicated a “probable case” of DIHS.^4^ Key features included lymphadenopathy, eosinophilia, extensive rash with characteristic DIHS findings, and exclusion of alternative diagnoses.

Initial management included oral prednisone 40 mg daily, hydrocortisone 2.5% cream to the face, and triamcinolone acetonide 0.1% ointment to the body twice daily as needed. On hospital day 2, there was partial improvement, although edema around the ears and cervical lymphadenopathy persisted, and her eosinophil count continued to rise. At that time, her prednisone increased to 60 mg daily. After 5 days of hospitalization, there was significant improvement, and the patient was discharged.

At dermatology follow-up 5 days postdischarge, her symptoms had largely resolved aside from mild facial edema. Repeat labs revealed normal absolute eosinophils (0.30 10∗3/uL) and ALT (20 U/L). Her prednisone was tapered to 50 mg, decreasing by 10 mg every 10 days.

## Discussion

This case illustrates a severe cutaneous adverse reaction associated with an over-the-counter supplement marketed as a GLP-1 mimetic. The patient’s clinical presentation, temporal relationship to *G sylvestre* use, laboratory abnormalities, and histopathology supported a diagnosis of DIHS. The absence of other new medications, identifiable infectious triggers, and clinical improvement following supplement discontinuation and systemic corticosteroid therapy strengthened the causal association.

DIHS is a rare, potentially life-threatening hypersensitivity reaction that typically presents 2 to 8 weeks after exposure to an offending agent.^5^^,^^6^ DIHS presents with fever, morbilliform eruption, facial edema, hematologic abnormalities, and multiple end-organ involvement, most commonly hepatitis.^5^ The estimated incidence is 1 case per 10,000 exposures, though under-reporting may lead to underestimation.^5^ While DIHS most frequently occurs in response to anticonvulsants and antibiotics, cases associated with natural supplements have also been reported.^4^, ^5^, ^6^, ^7^

*G sylvestre* extract is 1 such over the counter “natural” supplement marketed for glycemic control and weight loss. Its constituents, including gymnemic acids, saponins, and alkaloids, reportedly modulate incretin-related pathways and may interact with GLP-1 receptor signaling.^3^ However, these compounds possess immune-adjuvant and hepatotoxic potential, and long-term safety data are limited.^3^ The association of DIHS with *G sylvestre* or GLP-1 mimetic formulations is not well-documented.

This case highlights several important diagnostic considerations. DIHS should remain in the differential for patients presenting with a diffuse morbilliform eruption, facial edema, eosinophilia, and elevated liver enzymes, even in the absence of prescription drug exposure. Histopathologic findings in DIHS are often variable and nonspecific and largely reflect the observed clinical morphology. Reported reaction patterns may vary from spongiosis, subcorneal pustular dermatitis, or interface dermatitis.^8^ Prompt recognition, withdrawing the offending agent, and initiating systemic corticosteroids remain the cornerstone of management and are often effective when instituted early.^5^^,^^6^

*G sylvestre* or other ingredients in over-the-counter supplements are possible triggers of severe cutaneous adverse reactions which underscore the importance of including detailed supplement histories in dermatologic and inpatient evaluations. The growing use of unregulated “natural” products marketed to mimic GLP-1 agonists introduces new potential for immune-mediated toxicity, particularly in genetically or immunologically susceptible individuals. As consumer-available weight loss products continue to expand, dermatologists should remain vigilant for unanticipated reactions that mimic traditional drug-related hypersensitivity syndromes.

Our case contributes to the limited but growing evidence that over-the-counter supplements can induce systemic hypersensitivity reactions that constitute dermatologic emergencies. Increased awareness and reporting of such events are essential to improve regulatory oversight and patient safety.

## Conflicts of interest

None disclosed.
